# P-1267. Neisseria gonorrhoeae in Ecuador: insights from whole-genome sequencing

**DOI:** 10.1093/ofid/ofaf695.1457

**Published:** 2026-01-11

**Authors:** Jeannete Zurita, Fernando Lara-Freire, Gabriela Sevillano, Gabriela Sevillano, Andrés Herrera-Yela, Heydi Tonguino, Ariane Paz y Miño, Camilo Zurita-Salinas

**Affiliations:** Unidad de Investigaciones en Biomedicina. Zurita & Zurita Laboratorios, Quito, Pichincha, Ecuador; Biomedical Research Unit. Zurita & Zurita Laboratorios, Quito, Pichincha, Ecuador; Unidad de Investigaciones en Biomedicina. Zurita & Zurita Laboratorios, Quito, Pichincha, Ecuador; Unidad de Investigaciones en Biomedicina. Zurita & Zurita Laboratorios, Quito, Pichincha, Ecuador; Universidad Internacional SEK, Quito, Pichincha, Ecuador; Biomedical Research Unit. Zurita & Zurita Laboratorios, Quito, Pichincha, Ecuador; Mass General Brigham Salem Hospital, Salem, Massachusetts; Unidad de Investigaciones en Biomedicina. Zurita & Zurita Laboratorios, Quito, Pichincha, Ecuador

## Abstract

**Background:**

*Neisseria gonorrhoeae* causes over 80 million global gonorrhea cases annually and is increasingly resistant to first-line antibiotics. In Ecuador, genomic data is scarce due to empirical treatment practices and limited culture testing. This study aims to evaluate virulence and antimicrobial resistance (AMR) factors in *N. gonorrhoeae* strains from Quito, Ecuador.

**Figure:**
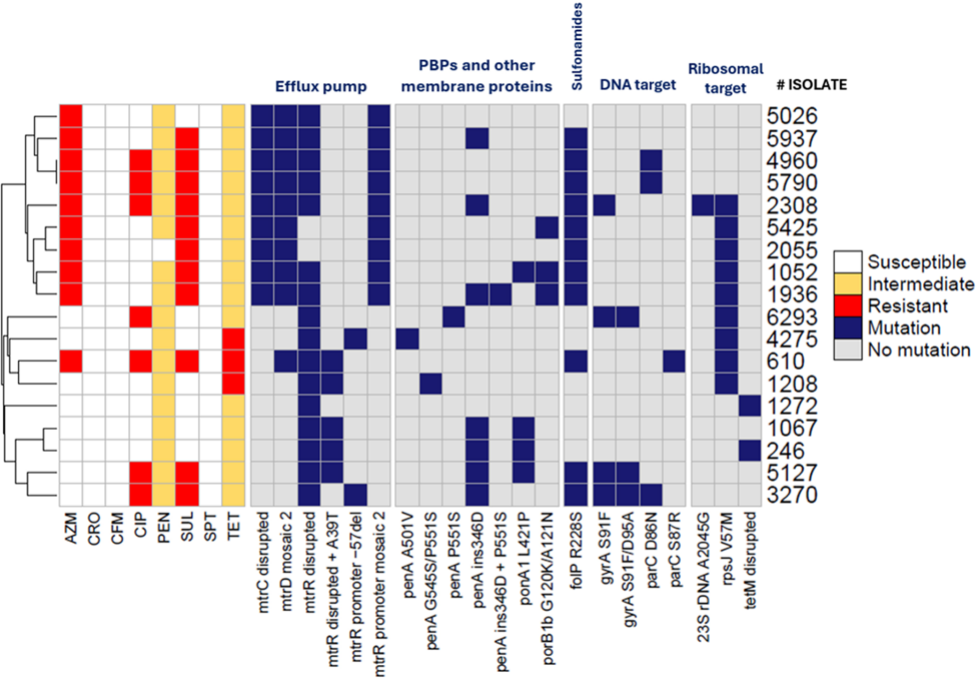
Antimicrobial resistance profiles in Neisseria gonorrhoeae strains.

**Figure:**
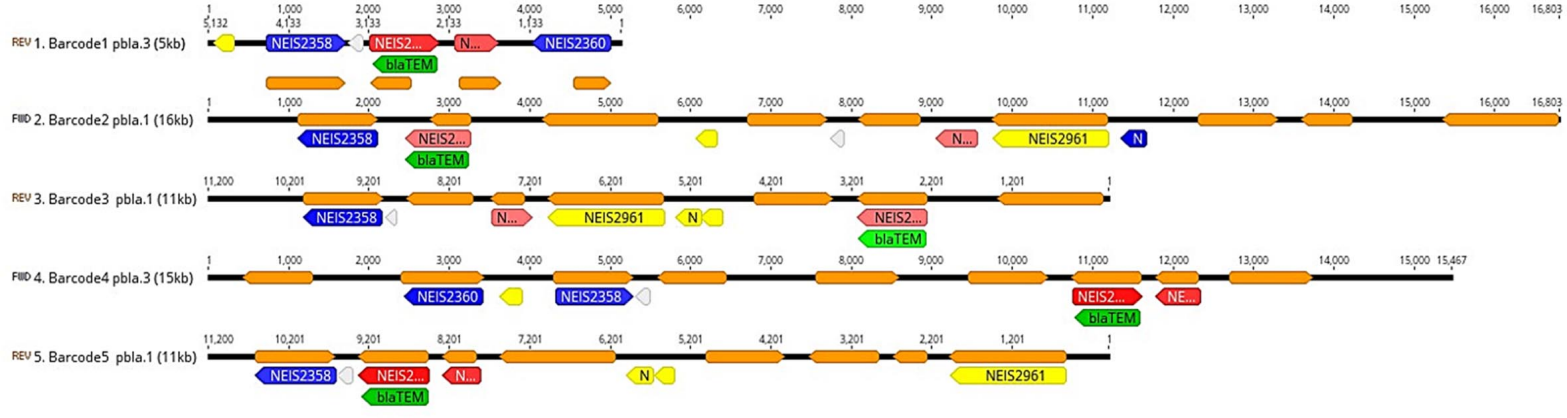
Schematic representation of the pbla.1 and pbla.3 associated to blaTEM. Grey line indicates aligned regions with arrows representing ORFs colored according to their gene products; grey, hypothetical proteins; yellow, mobilization proteins; red, Tn2-derived genes including blaTEM (NEIS2357); blue, replication initiation proteins.

**Methods:**

Twenty-five genital samples from adults with suspected gonorrhea (2021–2024) were cryopreserved. Eighteen isolates were successfully cultured and fully sequenced using Oxford Nanopore MinION™ MK1B. AMR profiles were analyzed with Pathogenwatch. The *bla*TEM gene was identified via sequence alignment and variant analysis. Plasmid-associated genes and replication/mobilization regions were characterized, and plasmid types were classified using a *pbla* typing scheme.

**Results:**

All isolates carried key virulence genes involved in adherence, invasion, and survival, including *pil*, *por*B, *kat*A, *Msr*A/B, *tbp*A/B, and *lbp*A/B. Multiple AMR mutations were detected (Figure 1). Five strains harbored the *bla*TEM gene, associated with plasmid variants *pbla.*1 (*bla*TEM-135) and *pbla*.3 (*bla*TEM-1B) (Figure 2).

**Conclusion:**

The detection of virulence and resistance genes in *N. gonorrhoeae* highlights its adaptability and the need for genomic surveillance. Ceftriaxone and cefixime remain effective for empirical treatment, but other antibiotics require prior sensitivity testing. Distinct features of *pbla.*1 and *pbla.*3 plasmids may affect gene transfer, stability, and spread. The presence of *bla*TEM in these plasmids raises concerns about potential extended-spectrum β-lactamases (ESBL) development, emphasizing the importance of tracking plasmid diversity to manage antibiotic resistance.

**Disclosures:**

All Authors: No reported disclosures

